# Recovering from depression with repetitive transcranial magnetic stimulation (rTMS): a systematic review and meta-analysis of preclinical studies

**DOI:** 10.1038/s41398-020-01055-2

**Published:** 2020-11-10

**Authors:** Luisa De Risio, Marta Borgi, Mauro Pettorruso, Andrea Miuli, Angela Maria Ottomana, Antonella Sociali, Giovanni Martinotti, Giuseppe Nicolò, Simone Macrì, Massimo di Giannantonio, Francesca Zoratto

**Affiliations:** 1 Department of Psychiatry and Addiction, ASL Roma 5, Colleferro, Rome, Italy; 2 Center for Behavioral Sciences and Mental Health, Istituto Superiore di Sanità, Rome, Italy; 3 Department of Neuroscience, Imaging and Clinical Sciences, “G. d’ Annunzio” University, Chieti, Italy; 4 Department of Pharmacy, Pharmacology, Clinical Science, University of Hertfordshire, Herts, UK

**Keywords:** Depression, Neuroscience

## Abstract

Repetitive transcranial magnetic stimulation (rTMS) has gained growing interest for the treatment of major depression (MDD) and treatment-resistant depression (TRD). Most knowledge on rTMS comes from human studies as preclinical application has been problematic. However, recent optimization of rTMS in animal models has laid the foundations for improved translational studies. Preclinical studies have the potential to help identify optimal stimulation protocols and shed light on new neurobiological-based rationales for rTMS use. To assess existing evidence regarding rTMS effects on depressive-like symptoms in rodent models, we conducted a comprehensive literature search in accordance with PRISMA guidelines (PROSPERO registration number: CRD42019157549). In addition, we conducted a meta-analysis to determine rTMS efficacy, performing subgroup analyses to examine the impact of different experimental models and neuromodulation parameters. Assessment of the depressive-like phenotype was quite homogeneous whilst rTMS parameters among the 23 included studies varied considerably. Most studies used a stress-induced model. Overall, results show a largely beneficial effect of active rTMS compared to sham stimulation, as reflected in the statistically significant recovery of both helplessness (SDM 1.34 [1.02;1.66]) and anhedonic (SDM 1.87 [1.02;2.72]) profiles. Improvement of the depressive-like phenotype was obtained in all included models and independently of rTMS frequency. Nonetheless, these results have limited predictive value for TRD patients as only antidepressant-sensitive models were used. Extending rTMS studies to other MDD models, corresponding to distinct endophenotypes, and to TRD models is therefore crucial to test rTMS efficacy and to develop cost-effective protocols, with the potential of yielding faster clinical responses in MDD and TRD.

## Introduction

Non-invasive brain stimulation (NIBS) uses magnetic pulses or weak electric currents to induce changes in cortical excitability and modulate brain networks in localized areas. Repetitive Transcranial Magnetic Stimulation (rTMS) is a neuromodulation technique that uses a magnetic field to stimulate focal cortical brain regions with electrical currents. Recently, rTMS has gained growing interest for the treatment of major depression (MDD) and treatment resistant depression (TRD). MDD is a highly prevalent psychiatric disorder that severely impairs functioning and diminishes quality of life. The global prevalence of MDD increased by almost 13% during 2007–2017^1^. Also, relapse rates are higher than for any other medical condition^2^. Core symptoms of major depressive episodes include depressed mood, decreased drive, loss of interest and pleasure. Numerous accessory symptoms co-occur and illness course may vary significantly (i.e., singular, recurring or chronic), defining different levels of depression severity. As a result, MDD is a highly heterogeneous syndrome encompassing varied symptom clusters and divergent treatment responses. 30–50% of patients do not adequately respond to first-line treatments, which generally involve a combination of antidepressant medication and cognitive-behavioral therapy^3^. Conventional antidepressants target the main neurotransmitter systems thought to be implicated in MDD (e.g., serotonin, noradrenaline, dopamine) and are associated with considerable variations in efficacy. A recent, comprehensive and large-scale meta-analysis revealed that antidepressants are generally more effective than placebo, although the overall effect size (0.3) is modest^4^. Treatment resistant depression (TRD) is defined as the absence of a clinical response despite at least 2 consecutive antidepressant trials (at adequate doses for at least 4–6 weeks). TRD is a major public health concern; functional impairment is greater and suicide risk is higher^5^. Despite the promising recent FDA approval of esketamine for TRD, there is a clear unmet need for rapidly-acting and efficacious treatments^6,7^.

The effects of neuromodulation produce a dynamic regulation of brain circuitry rather than directly affect neurotransmission. During rTMS, magnetic pulses are delivered by coils of different shapes (planar, figure-of-eight, helmets) at different frequencies (usually between 1 and 20 Hz) and intensities to determine changes in the excitability of specific brain areas. rTMS induces lasting changes in cortical excitability^8^. Repeated low-frequency stimulation (1 Hz) and the continuous form of theta-burst stimulation (cTBS) induce a suppression of excitatory synaptic transmission, while high-frequency stimulation (regular 5–50 Hz) and the intermittent form of theta-burst stimulation (iTBS) potentiate it^9,10^. In light of its effectiveness, rTMS is recommended by CANMAT guidelines as a first-line intervention after failure of one adequate antidepressant trial^11^. Evidence from meta-analyses suggests that rTMS has a comparable effect to ECT and antidepressant medication^12^. Recently, rTMS has been approved by the FDA for TRD. In the clinical setting, converging evidence supports a relevant role of physical therapies to treat TRD, mainly in the framework of integrated approaches, with response rates between 30 and 64% observed after rTMS interventions. Besides, rTMS is supposed to be a potential therapeutic option for substance use disorders (e.g.,^13,14^). Usually, rTMS protocols for MDD deliver 10 Hz stimulation of the left dorsolateral prefrontal cortex (DLPFC) at an intensity of 120% of the resting motor threshold (RMT) over 4–6 weeks in once-daily stimulation sessions^15^. Other protocols are available (i.e., right DLPFC inhibition, medial PFC stimulation) and appear to reduce depressive symptoms. Though rTMS is a promising treatment option for MDD, clinical response is partial, highlighting the need for a more thorough understanding of MDD pathophysiology and of mechanisms implicated in rTMS therapeutic action.

Most knowledge on rTMS comes from clinical studies as application to animal models has been problematic^16^. A major setback is lack of specificity of stimulation targets. While in humans technological advances allow for a very high level of accuracy (resulting in an isolated stimulation of a specific region), difficulty in maintaining small-scale focus has slowed rTMS use in animal models^17^. This has hampered gaining of the necessary understanding of the neurobiological basis of rTMS to develop personalized interventions and to clarify which stimulation protocols (i.e., number of pulses, stimulation frequency, and intersession pauses) yield faster responses, allowing only for empirically-based treatment protocols (stimulation protocols applied in clinical trials present limited variability in terms of rTMS intervention characteristics). Recently, optimization of rTMS use in animal models (e.g., mechanical restraint vs. anesthetic use^18^) and development of smaller sized coils specifically designed for preclinical application^19^ have laid the foundations for improved translational studies.

Numerous preclinical approaches have been developed over the years to model aspects of MDD in rodents^20,21^. The chronic unpredictable mild stress (CUS or CMS or CUMS; hereinafter referred to as CUS) is one of the most extensively investigated models^22^. It involves continuous exposure over several weeks to a variety of mild manipulations acting as low-grade stressors, determining induction of depressive-like symptoms, such as anhedonia (i.e., loss of pleasure for natural rewards), commonly measured by the sucrose preference test (SPT). This model simulates other phenotypic alterations isomorphic to human MDD symptoms, such as increased immobility in the forced swim test (FST) and changes in sleep architecture and locomotor activity^22^. Preclinical studies have the potential to shed light on new neurobiological-based rationales for rTMS use and to help identify optimal stimulation protocols (i.e., number of pulses, stimulation frequency and intersession pauses).

In order to assess the current status of translational application of rTMS in the preclinical field as a treatment for MDD, we systematically reviewed studies using rTMS in rodent models. We included studies applying rTMS to both animal models of depression and healthy animals that assessed changes in terms of depressive-like measures. In addition, we conducted a meta-analysis on the efficacy of rTMS treatment for recovery from the depressive phenotype, analyzing the possible impact of different experimental models and neuromodulation protocols on treatment outcome. Data are discussed to elucidate the translational relevance of preclinical findings in developing effective treatments for MDD and TRD.

## Methods

### Review protocol

The systematic search was conducted in accordance with the Preferred Reporting Items for Systematic Reviews and Meta-Analyses (PRISMA) guidelines^23,24^. The protocol (using SYRCLE’s systematic review protocol format for animal intervention studies^25^; Supplementary item 1) was submitted to the PROSPERO registry on November 6th, 2019 and registered on November 29th, 2019 (registration number: CRD42019157549).

### Literature search and study identification

A systematic literature search was conducted by comprehensive searches in three online databases (PubMed, Scopus, Web of Science). The search strategy consisted of two main components: repetitive transcranial magnetic stimulation (rTMS) and depression, and results were limited to rats and mice studies (as, together with fish, they are the main species used for scientific purposes in Europe^26,27^). The complete search strategies used in each database are presented in the supplementary material (Supplementary item 2). Searches were conducted on November 11th, 2019.

The following prioritization of exclusion criteria was used for both the 1st (i.e., titles and abstracts) and the 2nd (i.e., full-text articles) screening phases: (1) language other than English; (2) non-original researches (e.g., reviews, commentaries, editorials, book chapters); (3) no full-text articles (e.g., meeting abstracts); (4) studies in vitro, studies in humans, studies in non-human animals other than rodents; (5) other outcome measures reported (e.g., anxiety) in the absence of an assessment of the depressive-like phenotype; (6) neuromodulation interventions other than rTMS (e.g., transcranial direct current stimulation, tDCS); (7) animals not exposed to the sham rTMS intervention as comparator/control. Within each phase, two independent reviewers screened each article (AO, FZ), with discrepancies being resolved through discussion or by consulting additional investigators (MP, MB).

### Data extraction and synthesis

#### Qualitative synthesis

The full-text articles of studies eligible for qualitative data extraction were independently assessed by multiple reviewers (AO, FZ for data regarding the animal model; AM, AS for data regarding the stimulation parameters), with discrepancies that could not be resolved by discussion being solved by consulting additional investigators (MP, MB). The data extracted included the following categories: (i) bibliographic details; (ii) animal model characteristics; (iii) study design characteristics; (iv) intervention characteristics. Detailed information on the study characteristics extracted within each category is reported in the protocol (Supplementary item 1). Our primary outcome measure was the variation of the depressive-like phenotype in subjects exposed to active rTMS compared with sham intervention. In particular, we retrieved data on the direction of the variation (i.e., recovery vs. deterioration, including the augmenting or antagonizing effects of concomitant pharmacological interventions) of the reported variables within each test at all reported timepoints (i.e., ongoing, short-term, long-term). Additional outcome measures (when available) were the variation of other behavioral phenotypes relevant to depression (i.e., anxiety, locomotion, body weight) resulting from active (vs. sham) rTMS intervention.

#### Quantitative synthesis (meta-analysis)

The studies included in the qualitative synthesis were also eligible for quantitative data extraction. Selected outcomes were the short-term variation (i.e., 24 h after the last rTMS session) of the anhedonic profile and of the helplessness profile. If the 24-h timepoint had not been collected, data closest to the last rTMS session were extracted. Statistical details to enable the computation of standardized effect sizes, namely number of animals, mean and standard deviation (SD), were independently extracted by multiple reviewers (AO, FZ) from the graphs using a digital screen ruler^28^.

Effect size calculations were based on the comparison between the group receiving active rTMS intervention and the control group (sham). The intervention effect for each individual treated-control comparison was expressed as standardized difference in means (SDM; difference in mean between treated and control groups on pooled SD). The individual SDMs were pooled to obtain an overall SDM and 95% confidence interval (95% CI; indicating a range within which it can be 95% certain that the true effect lies). Whenever a control group served more than one experimental group, we corrected the total number of control animals in the meta-analysis by dividing the number of animals in the control group by the number of intervention groups served^29^.

Heterogeneity among results was explored by conducting subgroup analyses by rTMS intervention’s frequency and by type of animal model. Nevertheless, as animal studies are usually rather heterogeneous with respect to numerous factors (e.g., species/strain, procedures, etc.^28,29^), a random-effect model was used to compute both the overall effect size and the separate effect sizes for the different subgroups, in order to take into account heterogeneity that cannot be explained. In the presence of one or two studies presenting characteristics that render them different from the others, a sensitivity analysis was performed excluding those studies from the meta-analysis.

We calculated the *I*^2^ statistic for each analysis as a measure of the proportion of the overall variation that is attributable to between-study heterogeneity^30,31^. Specifically, we considered an *I*^2^ of less than 40% as low, between 30 and 60% as moderate, between 50 and 90% as substantial, and between 75 and 100% as considerable^32^.

To assess potential publication bias, a funnel plot of study effect sizes against standard errors was visually inspected for asymmetry resulting from a relative lack of small studies with small effect sizes (i.e., those most likely to be non-significant and to remain unpublished). Asymmetry was also statistically tested with Egger’s bias test^33^ with *p* < 0.05 indicating asymmetry. Statistical analyses were performed using Comprehensive Meta-Analysis (CMA), version 3.0. Statistical significance was set at *p* < 0.05.

### Assessment of the risk of bias

To assess the internal validity/methodological quality of the included studies, we used the SYRCLE’s Risk of Bias (RoB) tool for animal studies, developed by Hooijmans and co-authors^34^ by adjusting the Cochrane’s RoB tool^35^ for aspects of bias that play a specific role in animal studies. The RoB tool for animal studies contains 10 entries related to selection bias, performance bias, detection bias, attrition bias, reporting bias and other biases. Two independent reviewers (AO, AM) performed the quality assessment of each article by independently assessing the criteria.

## Results

### Study selection

**Fig. 1:**
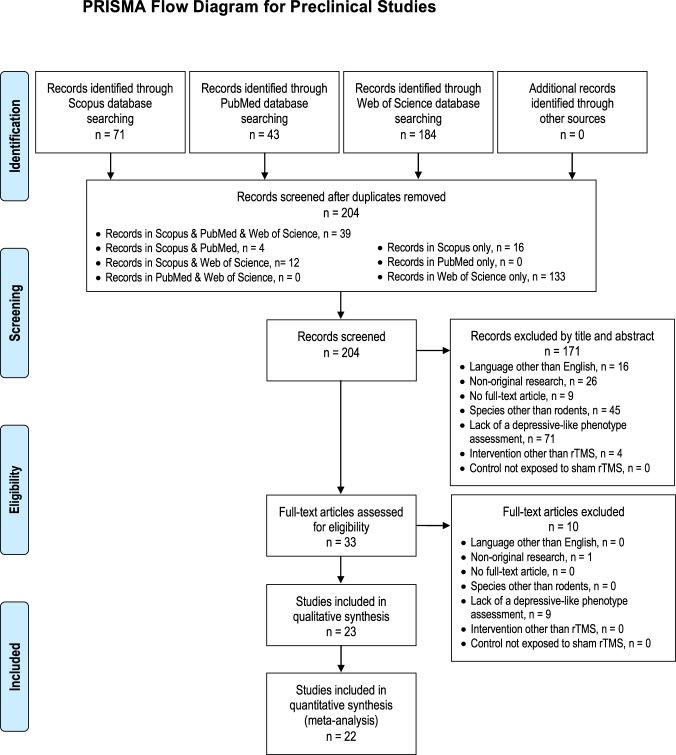
PRISMA flow diagram for preclinical studies^25^. Diagram of the literature search (identification) and selection process (screening, eligibility, inclusion).

The comprehensive search strategy on the effects of rTMS on depressive-like symptoms in rodent models resulted in 298 bibliographic records. The study selection process is summarized in Fig. 1 by using the PRISMA flow diagram. References were exported to Excel and, after duplicates were removed, 204 studies were left. The 1st selection phase (i.e., titles and abstracts screening) resulted in 33 studies; the 2nd selection phase (i.e., full-text articles screening) resulted in 23 studies eligible for inclusion in the systematic review, of which 22 could also be included in the meta-analysis (1 study^36^, was excluded as the number of animals in the control group after the required correction could not be processed by the CMA software).

### Study characteristics

The characteristics of the 23 included studies are described in Table 1. The assessment of the depressive-like phenotype was quite homogeneous; specifically, 15 articles^37–51^ investigated the helplessness profile through the Forced swim test (FST), 2 articles^52,53^ the anhedonic profile through the sucrose preference/consumption test (SPT/SCT) whilst the remaining 6 articles^36,54–58^ investigated both. In addition, the anxiety profile was assessed in 9 articles^37,40,46,47,51,54–56,58^ (by means of 5 different tests) and other behavioral profiles (i.e., general activity, weight measurement, social interaction, etc.) were assessed in 12 articles^36,37,40,42,45,46,51–53,56–58^.

**Table 1:** Characteristics of the included studies.

Study^a^		Animal model characteristics					rTMS intervention characteristics								Outcome			
		Species (strain), sex, weight/age	Model	Disease induction	N per group	Frequency (Hz)	N pulses per train	N trains	N sessions per intervention, inter-session interval	N pulses per intervention	Intensity^b^	Inter-train interval (s)	Coil type (ext. diam; int. diam); coil position	Drug combined with rTMS	Depression: helplessness	Depression: anhedonia	Anxiety	Other
Chen et al. 2015^54^		Rat (S-D), males, 180–220 g	D	CUS for a 4-week period	A: 12; S: 12	15	60	15	7 consecutive daily sessions	6300	1.26 T	15	n/a (5.0 cm; 2.5 cm); V	Quetiapine (10 mg/kg, i.p., 7 days)	X^c^	X^c^	X^c^	
Fang & Wang 2018^55^		Rat (S-D), males, 3 weeks	D	CUS for a 3-week period	A: 10; S: 10	15	60	15	7 consecutive daily sessions	6300	100% device max power	15	n/a (n/a; n/a); V	AM251 (1 mg/kg, i.p. and i.c., 7 days)	X^c^	X^c^	X^c^	
Feng et al. 2012^56^		Rat (S-D), males, 180–220 g/8 weeks	D	CUS for a 8-week period^g^	A: 12; S: 12	15	60	17	21 consecutive daily sessions	21000	100% device max power	15	F8 (7.0 cm; 5.0 cm); V	Venlafaxine (30 mg/kg, oral, 21 days)	X^d^	X^d^	X^d^	X^f^ (GA, AD)
Hargreaves et al. 2005^37^		Rat (S-D), males, 301 g/55 days	H	–	A: 12, S: 12	20	320	4	18 consecutive daily sessions	23040	74% device max power/130% MT	30	F8 (7.0 cm; 5.0 cm); SS		X^c^		X^c^	X^c^ (SI)
Heath et al. 2018^38^	a	Mice (C57), males, 8 weeks	D (model of agitated depression)	Olfactory bulbectomy (lesion)	A: 16; S: 12	10	n/a	n/a	20 sessions in 28 days (4 rounds of 5 days, with a 2-day interval)	36000	0.004 T	n/a	n/a (0.8 cm; n/a); FC		X^c^			
	b				A: 13; S: 12						0.05 T		n/a (0.8 cm; n/a); FC					
	c				A: 15; S: 12						1.0 T		C (4.0 cm; n/a); FC					
Hedges et al. 2003^44^		Rat (S-D), males, young adults	H	–	A: 6; S: 6	15	45	1	10 consecutive daily sessions	450	80% device max power	n/a	F8 (5.0 cm; n/a); 2 cm from V		X^d^			
Hesselberg et al. 2016^45^	a	Rat, males, 60–77 days	D	Flinders sensitive line (FSL) (genetic)	A: 10; S: 8	1	180	2	10 consecutive daily sessions	3600	110% MT	40	DC (2.5 cm; n/a); PFC		X^c^			X^c^ (GA)
	b					20												
Keck et al. 2000^46^		Rat (W), males, 42–58 g/4 weeks	H	–	A: 8; S: 12	20	50	3	25 sessions in 35 days (5 rounds of 5 days, with a 2-day interval)	3750	130% MT/4.0 T	120	C (5.7 cm; 0.6 cm); left FC		X^d^		X^c^	X^f^ (SI)
Keck et al. 2001^47^		Rat (W), males, 339–377 g/10 weeks	cD (anxiety)	High anxiety behavior (HAB) (selective breeding)	A: 11; S: 11	20	50	20	6 sessions in 10 days (2 rounds of 3 days, with a 4-day interval)	6000	130% MT/4.0 T	120	C (5.7 cm; 0.6 cm); left FC		X^c^		X^c^	
Kim et al. 2006^48^		Rat (S-D), males, 215–245 g	H	–	A: 9; S: 9	10	50	20	7 consecutive daily sessions	7000	1.4 T	25	F8 (7.0 cm; n/a); V		X^c^			
Kim et al. 2014^52^		Rat (S-D), males, 160–180 g	D	CUS for a 4-week period	A: 5; S: 5	10	50	20	14 consecutive daily sessions	14000	1.4 T	25	F8 (7.0 cm; n/a); FC			X^e^		X^e^ (W)
Peng et al. 2018^h^^36^	a	Rat (S-D), males, 180–220 g	D	CUS for a 4-week period	A: 10; S: 10	1	n/a	n/a	7 consecutive daily sessions	n/a	0.84 T	n/a	n/a (5.0 cm; 2.5 cm); V		X^c^	X^c^		X^c^ (GA)
	b					1					1.26 T							
	c					5					0.84 T							
	d					5					1.26 T							
	e					10					0.84 T							
	f					10					1.26 T							
Sachdev et al. 2002^49^	a	Rat (S-D), males, 200–250 g	H	–	A: 8; S: 8	1	n/a	n/a	5 consecutive daily sessions	5000	70% device max power/2.3 T	n/a	F8 (7.0 cm; n/a); n/a		X^c^			
	b					5												
	c					15												
	d					25												
Sun et al. 2011^50^		Mice (C57), males, 23–36 g/15–20 weeks	D	Modified version of forced swimming (5 days, 10 min per day)	A: 10; S: 9	10	50	1	28 consecutive daily sessions	1400	80% device max power	n/a	F8 (5.0 cm; n/a); B		X^c^			
Tan et al. 2018^51^		Rat (S-D), males/females, 30 days	cD (autism)	Neonatal isolation (postnatal day 1–9)	A: 19; S: 22	1	30	20	14 consecutive daily sessions	8400	50% device max power/100% MT	2	C (5.7 cm; 1.8 cm); 15 mm anterior to B		X^c^		X^c^	X^c^ (SI, S)
Tsutsumi et al. 2002^39^		Rat (W), males, 270–350 g/8 weeks	H	–	A: 10; S: 10	15	52	1	10 consecutive daily sessions	520	100% MT	n/a	C (4.0 cm; n/a); V		X^c^			
Wang et al. 2014^57^		Rat (S-D), males, 180–230 g	D	CUS for a 4-week period	A: 9; S: 9	15	60	15	7 consecutive daily sessions	6300	100% device max power	15	n/a (5.0 cm; 2.5 cm); V	AM251 (1 mg/kg, i.p., 7 days)	X^c^	X^c^		X^c^ (GA)
Wang et al. 2019^40^		Rat (S-D), males, 180–220 g/2 months	cD (epilepsy)	Injections of pentylenetetrazol (daily for 15 days)^g^	A: 12; S: 12	0.5	10	41	14 consecutive daily sessions	5740	n/a	2	C (5.7 cm; 1.8 cm); 15 mm anterior to B		X^c^		X^c^	X^c^ (GA)
Xue et al. 2019^58^	a	Rat (S-D), males, 280–320 g	D	CUS for a 4-week period	A: 12; S: 12	1	n/a	n/a	7 consecutive daily sessions	6300	1.26 T	8	C (5.0 cm; 2.5 cm); V		X^c^	X^c^	X^c^	X^c^ (GA)
	b					5	10	6		420								
Yang et al. 2007^41^		Rat (W), males/females, 180–220 g/10–12 weeks	H	–	A: 12; S: 12	15	50	4	10 consecutive daily sessions	2000	1.0 T	60	C (5.5 cm; n/a); V		X^c^			
Zhao et al. 2018^53^		Rat (S-D), males, 150–180 g	D	CUS for a 3-week period	A: 14; S: 14	10	10	50	15 sessions in 21 days (3 rounds of 5 days, with a 2-day interval)	7500	50% MT	10	C (5.0 cm; n/a); SS			X^a^		X^c^ (GA, W)
Zyss et al. 1997^42^		Rat (W), males, 180–220 g/2–3 months	H	–	A: 10; S: 10	50	15000	1	10 sessions in 14 days (2 rounds of 5 days, with a 2-day interval)	150000	0.1 T	n/a	n/a (n/a; n/a); n/a		X^c^			X^c^ (GA, A)
Zyss et al. 1999^43^	a	Rat (W), males, 280–330 g/3 months	H	–	A: 16; S: 24	20	6000	1	9 consecutive daily sessions	54000	1.6 T	n/a	C (n/a; n/a); V		X^c^			
	b				A: 16; S: 24	20	6000		18 consecutive daily sessions	108000								
	c				A: 8; S: 24	30	9990		9 consecutive daily sessions	89910								

*S-D* Sprague-Dawley strain, *W* Wistar strain, *D* depression, *cD* comorbid depression, *H* healthy, *CUS* chronic unpredictable stress, *A* active rTMS intervention, *S* sham rTMS intervention, *F8* figure eight/focal butterfly coil, *DC* double coil, *C* round/circular coil, *V* vertex of the skull, *PFC* prefrontal cortex, *FC* frontal cortex, *B* bregma, *SS* sagittal suture, *i.p.* intraperitoneal, *i.c.* intracerebral, *GA* general activity, *AD* appetitive drive, *SI* social interaction, *W* weight measurement, *S* stereotyped behaviors, *A* analgesia, *n/a* not available.

^a^Studies with multiple experimental groups (i.e., exposed to rTMS intervention with a different number of pulses per intervention or a different intensity) are split in multiple lines (indicated by a, b, c, etc.). ^b^Intensity could be either expressed as % device max power, % motor threshold (MT) or Tesla (T). Timing of the assessment (referred to the rTMS intervention): ^c^only short-term (<1 week after the last rTMS session); ^d^both short-term (<1 week after the last rTMS session) and long-term (>1 week after the last rTMS session); ^e^only ongoing; ^f^only long-term (>1 week after the last rTMS session). ^g^Procedure simultaneous with rTMS intervention (in all other articles, rTMS intervention entirely preceded by the disorder induction). ^h^Not included in the meta-analysis.

In 22 out of 23 articles at least one assessment of the depressive-like phenotype was performed shortly after the end of the rTMS intervention. Only in 1 article^52^ tests were performed only during the rTMS intervention (in this case the one closest to the end was selected). Specifically, for the short-term evaluation of rTMS efficacy (included in the meta-analysis), the timing of the tests relative to the neurostimulation intervention was as follows: 24 h after the last rTMS session (12 articles^36,38–44,47,54,55,57^); immediately after the last rTMS session (5 articles^37,48–50,53^); between 24 and 72 h after the last rTMS session (3 articles^45,46,58^); during the week following the end of the rTMS intervention (2 articles^51,56^); during the last week of the rTMS intervention (1 article^52^).

A number of articles also reported ongoing and long-term evaluations of rTMS efficacy performed at various additional timepoints (not included in the meta-analysis). Interestingly, 3 articles out of 23 extended the evaluation of rTMS efficacy to the long-term period (i.e., >1 week after the last rTMS session): 1 week and 2 weeks after the last session of a 10-days intervention^44^; during the 2nd week after a 5-weeks intervention^46^; during the 3rd week after a 3-weeks intervention^56^.

Treated subjects were either models of depression (11 articles^36,38,45,50,52–58^) or models of other disorders with comorbid depression (3 articles^40,47,51^) or healthy animal models (9 articles^37,39,41–44,46,48,49^) receiving active rTMS intervention; control subjects were either models of depression or animals modeling other disorders or healthy animals receiving the sham rTMS intervention, respectively. Regarding the models of depression, 8 articles^36,52–58^ employed the chronic unpredictable mild stress (CUS) model (4-week protocol in 5 articles^36,52,54,57,58^, 3-week protocol in 2 articles^53,55^, 8-week protocol in 1 article^56^); 1 article^50^ applied a modified version of a forced swimming paradigm (10 min daily for 5 days) able to induce a depression-like state durable for 4 weeks without additional swimming; 1 article^45^ employed a genetic model, i.e., the Flinders sensitive line (FSL) and its control (the Flinders resistant line, FRL); 1 article^38^ applied a lesion to obtain the olfactory bulbectomy model of agitated depression^59^. The models of comorbid depression were a model of anxiety from selective breeding^47^, a model of autism through neonatal isolation^51^ and a model of epilepsy by means of pentylenetetrazol administration^40^.

In 12 out of 14 articles employing a disease model, the rTMS intervention was entirely preceded by the disorder induction; in the remaining 2 articles the CUS procedure^56^ and the pentylenetetrazol injections^40^ were simultaneous with the rTMS intervention.

The neurostimulation parameters among studies varied considerably. The frequencies employed ranged from 0.5 Hz to 50 Hz (<5 Hz in 6 articles^36,40,45,49,51,58^, =5 Hz in 3 articles^36,49,58^, >5 Hz in 20 articles^36–39,41–50,52–57^; 5 studies employed more frequencies in distinct groups of animals^36,43,45,49,58^). The intensity could be either expressed as Tesla (from 0.004 to 4.0T), % motor threshold (MT; from 50 to 130%) and/or % device maximum power; 2 studies^36,38^ employed more intensities in distinct groups of animals and 1 study^40^ did not mention this parameter. Number of pulses per single session (i.e., N pulses per train × N trains) and total number of pulses administered during the entire intervention varied greatly, from 45 to 15,000 and from 420 to 150,000 respectively. When mentioned, the inter-train interval ranged from 2 to 120 s. The total number of sessions per intervention varied from 5 to 28 (≤7 in 8 articles^36,47–49,54,55,57,58^, >7 in 15 articles^37–46,50–53,56^). In general, the inter-session interval was 24 h (up to 72 h in the 4 studies that interrupted treatment for weekends^38,42,46,53^; up to 120 h in^47^) as no accelerated protocols were applied. Whilst 5 studies^36,42,54,55,57^ did not mention the type of coil, the remaining studies used 3 types of coil (for details about coil size and position see Table 1). Only 1 article used anesthesia^47^.

Only 2 articles used female subjects (pool of males and females^51^; comparison between males and females^41^), the remaining 21 articles employed only male subjects. Only 2 articles^38,50^ employed mice (C57 strain), the remaining 21 articles used rats (Sprague-Dawley or Wistar strains); age and/or weight were rather heterogeneous (for details see Table 1). Finally, only 4 articles^54–57^ evaluated the potential additive/antagonistic effects deriving from the concomitant administration of neuromodulation and pharmacological interventions (i.e., the atypical antipsychotic quetiapine^54^, the CB1 receptor antagonist AM251^55,57^, and the antidepressant venlafaxine^56^).

### rTMS efficacy on depressive-like symptoms

The effects of rTMS intervention on helplessness and anhedonia on all reported parameters (within each test) are illustrated in Table 2 and in Table 3, respectively. For the purpose of the systematic review, the tables include, under separate headings, not only the 24-h outcome (or the outcome closest to the last rTMS session), which was included in the meta-analysis, but also the outcomes from all reported timepoints (on-going, shorth-term, long-term).

**Table 2:** rTMS effects on the helplessness profile.

Study	Intervention duration	Included in the meta-analysis			Other assessments		
		Timing	Test: parameter(s) used	Results	Timing	Test: parameter(s) used	Results
Chen et al. 2015^54^	7 days	24 h after the last session	FST: immobility duration (s)	15 Hz: ↓ (=recovery)			
Fang & Wang 2018^55^	7 days	24 h after the last session	FST: immobility duration (s)	15 Hz: ↓ (=recovery)	24 h after the last session	FST: latency to immobility (s)	15 Hz: ↑ (=recovery)
						FST: swimming duration (s)	15 Hz: ↑ (=recovery)
Feng et al. 2012^56^	3 weeks	During the 1^st^ week after the intervention	FST: immobility duration (s)	15 Hz: ↓ (=recovery)	During the 1st week after the intervention	FST: climbing duration (s)	15 Hz: ↑ (=recovery)
					During the 3rd week after the intervention	FST: immobility duration (s)	15 Hz: ↓ (=recovery)
						FST: climbing duration (s)	15 Hz: ↑ (=recovery)
Hargreaves et al. 2005^37^	18 days	Immediately after the last session	FST: immobility duration (s)	20 Hz: ↓ (=improvement)	17 days after the first session	FST: immobility duration (s)	20 Hz: ns
Heath et al. 2018^38^	4 weeks	24 h after the last session	FST: immobility duration as % of the pre-surgery value	10 Hz (0.004 T): ns; 10 Hz (0.05 T): ns; 10 Hz (1.0 T): ↑ (=recovery^a^)			
Hedges et al. 2003^44^	10 days	24 h after the last session	FST: swimming duration (s)	15 Hz: ns	Immediately after the last session	FST: swimming duration (s)	15 Hz: ↑ (=improvement)
					3 days after the last session		15 Hz: ns
					5 days after the last session		15 Hz: ns
					7 days after the last session		15 Hz: ns
					14 days after the last session		15 Hz: ns
Hesselberg et al. 2016^45^	10 days	24/48 h after the last session	FST: immobility duration (s)	1 Hz: ↓ (=recovery); 20 Hz: ↓ (=recovery)	24/48 h after the last session	FST: struggling duration (s)	1 Hz: ↑ (=recovery); 20 Hz: ↑ (=recovery)
Keck et al. 2000^46^	5 weeks	48 h after the last session	FST: immobility duration (s)	20 Hz: ↓ (=improvement)	48 h after the last session	FST: struggling duration (s)	20 Hz: ↑ (=improvement)
						FST: latency to immobility (s)	20 Hz: ↑ (=improvement)
					During the 2nd week after the intervention	FST: immobility duration (s)	20 Hz: ↓ (=improvement)
						FST: struggling duration (s)	20 Hz: ↑ (=improvement)
						FST: latency to immobility (s)	20 Hz: ↑ (=improvement)
Keck et al. 2001^47^	10 days	24 h after the last session	FST: immobility duration (s)	20 Hz: ↓ (=recovery)	24 h after the last session	FST: struggling duration (s)	20 Hz: ↑ (=recovery)
						FST: latency to immobility (s)	20 Hz: ↑ (=recovery)
						FST: swimming duration (s)	20 Hz: ns
Kim et al. 2006^48^	7 days	Immediately after the last session	FST: immobility duration (s)	10 Hz: ↓ (=improvement)	Immediately after the first session	FST: immobility duration (s)	10 Hz: ns
Peng et al. 2018^b^^36^	7 days				24 h after the last session	FST: immobility duration (%)	1 Hz (0.84 T): ns; 1 Hz (1.26 T): ns; 5 Hz (0.84 T): ↓ (=recovery); 5 Hz (1.26 T): ↓ (=recovery); 10 Hz (0.84 T): ↓ (=recovery); 10 Hz (1.26 T): ↓ (=recovery)
Sachdev et al. 2002^49^	5 days	Immediately after the last session	FST: immobility duration (s)	1 Hz: ns; 5 Hz: ns; 15 Hz: ↓ (=improvement); 25 Hz: ↓ (=improvement)	12 h after the first session	FST: immobility duration (s)	1 Hz: ↓ (=improvement); 5 Hz: ↓ (=improvement); 15 Hz: ↓ (=improvement); 25 Hz: ↓ (=improvement)
					48 h after the last session		1 Hz: ns; 5 Hz: ns; 15 Hz: ns; 25 Hz: ↓ (=improvement)
					72 h after the first session		1 Hz: ↓ (=improvement); 5 Hz: ↓ (=improvement); 15 Hz: ↓ (=improvement); 25 Hz: ↓ (=improvement)
Sun et al. 2011^50^	4 weeks	Immediately after the last session	FST: immobility duration (s)	10 Hz: ↓ (=recovery)	Immediately after the last session	FST: distance traveled (m)	10 Hz: ↑ (=recovery)
Tan et al. 2018^51^	2 weeks	96 h after the last session	FST: struggling duration (s)	1 Hz: ↑ (=recovery)	96 h after the last session	FST: latency to immobility (s)	1 Hz: ↑ (=recovery)
Tsutsumi et al. 2002^39^	10 days	24 h after the last session	FST: motor activity	15 Hz: ↑ (=improvement)	24 h after the first session	FST: motor activity	15 Hz: ns
Wang et al. 2014^57^	7 days	24 h after the last session	FST: immobility duration (s)	15 Hz: ↓ (=recovery)			
Wang et al. 2019^40^	2 weeks	24 h after the last session	FST: immobility duration (s)	0.5 Hz: ↓ (=recovery)	8 days after the first session	FST: immobility duration (s)	0.5 Hz: ns
Xue et al. 2019^58^	7 days	24/72 h after the last session	FST: immobility duration (s)	1 Hz: ns; 5 Hz: ↓ (=recovery)			
Yang et al. 2007^41^	10 days	24 h after the last session	FST: immobility duration (s)	15 Hz: Males: ↓ (=improvement); Females: ↓ (=improvement)	24 h after the last session	FST: latency to immobility (s)	15 Hz: Males: ↑ (=improvement); Females: ↑ (=improvement)
						FST: climbing duration (s)	15 Hz: Males: ↓; Females: ns
						FST: swimming duration (s)	15 Hz: Males: ns; Females: ↑ (=improvement)
Zyss et al. 1997^42^	2 weeks	24 h after the last session	FST: immobility duration (s)	50 Hz: ↓ (=improvement)			
Zyss et al. 1999^43^	9/18 days	24 h after the last session	FST: immobility duration (% of control sham)	20 Hz (300 s, 9 sessions): ns; 20 Hz (300 s, 18 sessions): ↓ (=improvement); 30 Hz (333 s, 9 sessions): ↓ (=improvement)			

*Notes:* Timing is referred to the rTMS intervention; recovery: recovery of the phenotype in models of disease; improvement: improvement of the behavioral profile in healthy models.

*FST* forced swim test, ↓ ↑: statistically significant change, *ns* not significant.

^a^Only in this study an increase in immobility duration is indicative of recovery of the phenotype (attenuated psychomotor agitation). ^b^Not included in the meta-analysis.

**Table 3:** rTMS effects on the anhedonic profile.

Study	Intervention duration	Included in the meta-analysis			Other assessments		
		Timing	Test: parameter(s) used	Results	Timing	Test: parameter(s) used	Results
Chen et al. 2015^54^	7 days	24 h after the last session	SPT: sucrose preference index (0–1)	15 Hz: ↑ (=recovery)			
Fang & Wang 2018^55^	7 days	24 h after the last session	SPT: sucrose preference ratio (%)	15 Hz: ↑ (=recovery)			
Feng et al. 2012^56^	3 weeks	During the 1st week after the intervention	SPT: sucrose preference index (0–1)	15 Hz: ↑ (=recovery)	During the 3rd week after the intervention		15 Hz: ↑ (=recovery)
Kim et al. 2014^52^	2 weeks	During the 2nd week of intervention^a^	SPT: sucrose preference ratio (%)	10 Hz: ↑ (=recovery)	During the 1st week of intervention	SPT: sucrose preference ratio (%)	10 Hz: ↑ (=recovery)
			SPT: absolute sucrose intake (g)	10 Hz: ↑ (=recovery)		SPT: absolute sucrose intake (g)	10 Hz: ↑ (=recovery)
Peng et al. 2018^b^^36^	7 days				24 h after the last session	SPT: sucrose preference ratio (%)	1 Hz (0.84 T): ns; 1 Hz (1.26 T): ns; 5 Hz (0.84 T): ↑ (=recovery); 5 Hz (1.26 T): ↑ (=recovery); 10 Hz (0.84 T): ↑ (=recovery); 10 Hz (1.26 T): ↑ (=recovery)
Wang et al. 2014^57^	7 days	24 h after the last session	SPT: sucrose preference index (0–1)	15 Hz: ↑ (=recovery)			
Xue et al. 2019^58^	7 days	24/72 h after the last session	SPT: sucrose preference ratio (%)	1 Hz: ns; 5 Hz: ↑ (=recovery)			
Zhao et al. 2018^53^	3 weeks	Immediately after the last session	SCT: sucrose intake (ml/100 g)	10 Hz: ↑ (=recovery)			

*Notes:* Timing is referred to the rTMS intervention; recovery: recovery of the phenotype in animal models of disease.

*SPT* sucrose preference test, *SCT* sucrose consumption test, ↑: statistically significant change, *ns* not significant.

^a^In all studies except this one at least 1 assessment was performed shortly after the end of the rTMS intervention; as in this study by assessments were only performed during rTMS intervention, the one closest to the end of the intervention was selected for the inclusion in the meta-analysis. ^b^Not included in the meta-analysis.

### Quantitative analysis of rTMS efficacy on the helplessness profile

Twenty studies (29 independent comparisons) measured the short-term efficacy of rTMS on the immobility/activity duration in the FST (Table 2). It should be noted that the recovery of the depressed phenotype in all studies but one corresponds to decreased immobility or increased activity in the FST; only in the model of agitated depression^38^ the recovery corresponds to increased immobility duration. Overall, rTMS led to a significant recovery of the phenotype in models of disease or improvement of the behavioral profile in healthy models (322 treated animals, 232 control animals; SDM = 1.34; CI 95%: 1.02–1.66; *Z* = 8.21, *p* < 0.001; Supplementary item 3). Between-study heterogeneity (*I*^2^) was 60%.

**Fig. 2:**
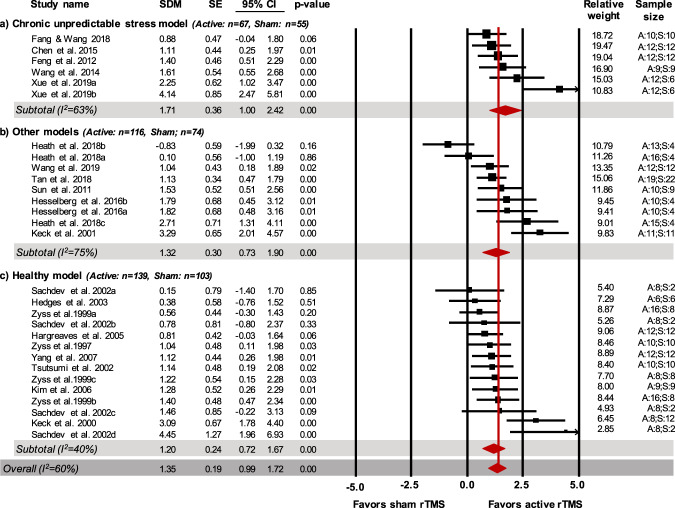
Forest plot (effect size and 95% CI) of individual comparisons of animals receiving active vs. sham rTMS intervention on the helplessness profile for subgroup analyses based on animal models. **a** chronic unpredictable stress model; **b** other models; **c** healthy model. *Notes*. Horizontal lines represent 95% CIs. The area of each square is proportional to the study weight in the analysis. The diamond represents pooled estimates from random-effects meta-analysis. Red line represents the overall effect. Studies with multiple experimental groups (i.e., exposed to rTMS intervention with a different number of pulses per intervention or a different intensity) are split in multiple lines (indicated by a, b, c, d); these were considered as independent comparisons in the meta-analysis after correcting the total number of control animals by dividing the number of animals in the control group by the number of intervention groups served. A: active rTMS intervention; S: sham rTMS intervention; SDM: standardized mean difference; SE: standard error; CI: confidence interval.

Eleven studies (15 comparisons) assessed the effects of rTMS in animal models of disease: 5 studies (6 comparisons) in the chronic unpredictable stress model of depression and 6 studies (9 comparisons) in other models of depression; the latter subgroup included 3 studies (3 comparisons) in models of other disorders with comorbid depression. In the remaining 9 studies (14 comparisons) rTMS effects were evaluated in healthy models. Recovery/improvement in the helplessness profile was observed in the CUS model (67 treated animals, 55 control animals; SDM = 1.71; CI 95%: 1.00–2.42; *Z* = 4.73; *p* < 0.001, *I*^2^ = 63%), in other models (116 treated animals, 74 control animals; SDM = 1.32; CI 95%: 0.73–1.90; *Z* = 4.42, *p* < 0.001, *I*^2^ = 75%) and in healthy models (139 treated animals, 103 control animals; SDM = 1.20; CI 95%: 0.72–1.67; *Z* = 4.90; *p* < 0.001, *I*^2^ = 40%; Fig. 2).

**Fig. 3:**
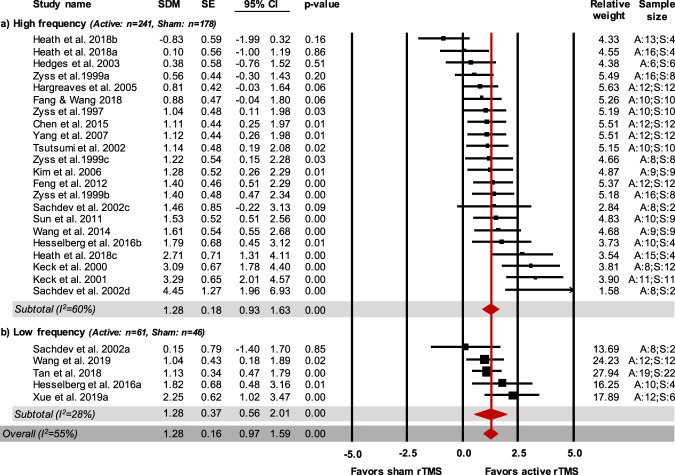
Forest plot (effect size and 95% CI) of individual comparisons of animals receiving active vs. sham rTMS intervention on the helplessness profile for subgroup analyses based on rTMS frequency. **a** high: >5 Hz; **b** low: <5 Hz (excluding = 5 Hz, i.e., Xue et al. 2019b, Sachdev et al. 2002b). *Notes*. Horizontal lines represent 95% CIs. The area of each square is proportional to the study weight in the analysis. The diamond represents pooled estimates from random-effects meta-analysis. Red line represents the overall effect. Studies with multiple experimental groups (i.e., exposed to rTMS intervention with a different number of pulses per intervention or a different intensity) are split in multiple lines (indicated by a, b, c, d); these were considered as independent comparisons in the meta-analysis after correcting the total number of control animals by dividing the number of animals in the control group by the number of intervention groups served. A: active rTMS intervention; S: sham rTMS intervention; SDM: standardized mean difference; SE: standard error; CI: confidence interval.

In 17 of the selected studies (22 comparisons) rTMS was given at high frequency (>5 Hz), in 5 studies (5 comparisons) at low frequency (<5 Hz) and in 2 studies (2 comparisons) at 5 Hz. The latter were excluded as (i) they were not enough to create an additional subgroup, (ii) they could not be attributed to either the low or the high frequency subgroups^16,60^. Recovery/improvement in the helplessness profile was observed in animals treated at high frequency (241 treated animals, 178 control animals; SDM = 1.28; CI 95%: 0.93–1.63; *Z* = 7.21; *p* < 0.001, *I*^2^ = 60%) and in animals treated with rTMS at low frequency (61 treated animals, 46 control animals; SDM = 1.28; CI95%: 0.56–2.01; *Z* = 3.46; *p* = 0.001, *I*^2^ = 28%; Fig. 3).

A sensitivity analysis, performed excluding 3 comparisons presenting characteristics that render them different from the others (anesthesia:^47^; particularly high frequencies, i.e., 50 Hz and 30 Hz:^42,43^), confirmed the beneficial effect of rTMS on helplessness profile (293 treated animals, 203 control animals; SDM = 1.29; CI 95%: 0.96–1.62; *Z* = 7.61, *p* < 0.001, *I*^2^ = 58%).

Inspection of the funnel plot of study effect sizes (SDMs) against standard errors (Supplementary item 4a) suggested asymmetry. Specifically, the funnel plot shows larger studies (smaller SE, appearing towards the top of the graph) clustered near the mean effect size, while smaller studies (higher SE, appearing towards the bottom of the graph) more dispersed across a wider range of values; the graph also shows a lack of small studies with small effect sizes. Egger’s test confirmed asymmetry that was consistent with publication bias (*p* = 0.001).

### Quantitative analysis of rTMS efficacy on the anhedonic profile

**Fig. 4:**
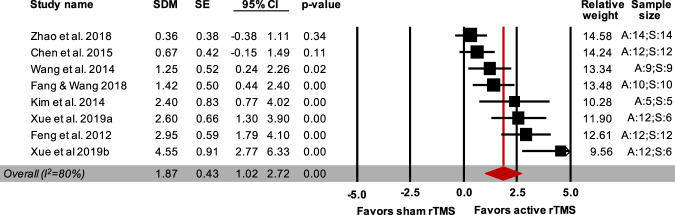
Forest plot (effect size and 95% CI) of individual comparisons of animals receiving active (*n* = 86 animals) vs. sham (*n* = 74 animals) rTMS intervention on the anhedonic profile (overall effect). *Notes*. Horizontal lines represent 95% CIs. The area of each square is proportional to the study weight in the analysis. The diamond represents pooled estimates from random-effects meta-analysis. Red line represents the overall effect. Studies with multiple experimental groups (i.e., exposed to rTMS intervention with a different number of pulses per intervention or a different intensity) are split in multiple lines (indicated by a, b, c, etc.); these were considered as independent comparisons in the meta-analysis after correcting the total number of control animals by dividing the number of animals in the control group by the number of intervention groups served. A: active rTMS intervention; S: sham rTMS intervention; SDM: standardized mean difference; SE: standard error; CI: confidence interval.

Seven studies (8 independent comparisons) measured the short-term efficacy of rTMS on the sucrose preference index/ratio in the SPT or sucrose intake in the SCT (Table 3). Overall, rTMS led to a significant improvement in the anhedonic profile (86 treated animals, 74 control animals; SDM = 1.87; CI 95%: 1.02–2.72; *Z* = 4.30, *p* < 0.001; Fig. 4). Between-study heterogeneity (*I*^2^) was 80%.

Inspection of the funnel plot of study effect sizes (SDMs) against standard errors (Supplementary item 4b) suggested asymmetry. Specifically, the top of the graph (larger studies, smaller SE), shows a higher concentration of studies on the left side of the mean effect size (i.e., smaller effects or no effects) while the bottom of the funnel plot (smaller studies, higher SE) shows a higher concentration of studies on the right side of the mean effect size (i.e., larger effects). Egger’s test confirmed asymmetry that was consistent with publication bias (*p* = 0.001).

### Other considerations

Regarding the long-term efficacy, 2 studies reported persistent beneficial effects on depressive-like symptoms during the 2nd week after a 5-weeks intervention^46^ and during the 3rd week after a 3-weeks intervention^56^. By contrast, 1 study did not detect a significant improvement neither 1 week nor 2 weeks after a 10-days intervention^44^.

As for the augmenting or antagonizing effects of concomitant pharmacological interventions, 1 study reported an additive effect of rTMS and the atypical antipsychotic quetiapine on both helplessness (compared with quetiapine alone) and anhedonia (compared with both rTMS alone and quetiapine alone)^54^; 2 studies reported an antagonizing effect of rTMS and the CB1 receptor antagonist AM251 (by either i.p. or intra-hippocampal injections) on both helplessness (compared with rTMS alone) and anhedonia (compared with rTMS alone)^55,57^. Finally, although the rTMS intervention produced antidepressant effects similar to those of the antidepressant venlafaxine on both helplessness and anhedonia, the combination of the 2 interventions had no additive effect compared with either rTMS or venlafaxine alone^56^.

### rTMS efficacy on other behavioral phenotypes relevant to depression

The effects of rTMS intervention on anxiety and other profiles on all available parameters (within each test) and timepoints (on-going, shorth-term, long-term) are illustrated in Table 4 and in Supplementary item 5, respectively.

**Table 4:** rTMS effects on the anxiety-like profile.

Study	Intervention duration	Test: parameter(s) used	1st assessment		2nd assessment	
			Timing	Results	Timing	Results
Chen et al. 2015^54^	7 days	OFT: time in the center (%)	24 h after the last session	ns		
Fang & Wang 2018^55^	7 days	NSFT: latency to feed (s)	24 h after the last session	↓ (=recovery)		
Feng et al. 2012^56^	3 weeks	NSFT: latency to feed (s)	During the 1st week after the intervention	↓ (=recovery)	During the 3rd week after the intervention	↓ (=recovery)
Hargreaves et al. 2005^37^	18 days	EPMT: time on open arms (%)	11 days after the first session	ns		
		Emergence test: latency to leave the box (s)	10 days after the first session	ns		
		Emergence test: risk assessment duration (s)		ns		
		Emergence test: time in open arena (s)		ns		
		Predator odor avoidance test: time in hide box (s)	14 days after the first session	ns		
		Predator odor avoidance test: Risk-assessment duration (s)		ns		
		Predator odor avoidance test: Time close to the collar (s)		ns		
Keck et al. 2000^46^	5 weeks	EPMT: time on open arms (s)	24 h after the last session	ns		
		EPMT: latency to enter open arms (s)		ns		
Keck et al. 2001^47^	10 days	EPMT: time on open arms (%)	24 h after the last session	ns		
		EPMT: entries into open arms (%)		ns		
Tan et al. 2018^51^	2 weeks	EPMT: time on open arms (s)	72 h after the last session	↑ (=recovery)		
		EPMT: entries into open arms		↑ (=recovery)		
Wang et al. 2019^40^	2 weeks	EPMT: time on open arms (%)	8 days after the first session	ns	24 h after the last session	↑ (=recovery)
		EPMT: entries into open arms (%)		ns		↑ (=recovery)
Xue et al. 2019^58^	7 days	OFT: time in the center (%)	24/72 h after the last session	1 Hz: ns; 5 Hz: ↑ (=recovery)		
		OFT: distance traveled in the center (%)		1 Hz: ns; 5 Hz: ↑ (=recovery)		

*Notes:* Timing is referred to the rTMS intervention; recovery: recovery of the phenotype in animal models of disease.

*OFT* open-field test, *NSFT* novelty-suppressed feeding test, *EPMT* elevated plus-maze test, ↓ ↑: statistically significant change, *ns* not significant.

### Anxiety

The effects of rTMS intervention on the anxiety profile were mixed (Table 4); a beneficial effect was reported in 2 studies out of 5 in the Elevated plus-maze test (EPMT), as evidenced by the increased time spent on open arms^40,51^, in 1 study out of 2 in the Open-field test (OPT), in terms of increased time spent in the center^58^, and in the 2 studies employing the Novelty-suppressed feeding test (NSFT), as evidenced by the decreased latency to feed^55,56^. Interestingly, the rTMS intervention did not ameliorate symptoms in the model of anxiety^47^, in spite of the recovery of the comorbid depressive-like profile.

### Other domains

As for the effects of the neurostimulation intervention on general activity, 3 studies out of 8 reported an increase in distance traveled in the OFT (Supplementary item 5, which also contains a comment on the potential confounding effects due to changes in locomotor activity in the interpretation of the readouts used to assess the effectiveness of rTMS). Although the rTMS intervention had no effect on social interactions in 2 studies employing healthy models, an increased sociality was reported in a model of autism^51^. In 2 studies the treatment attenuated the weight reduction induced by the CUS procedure^52,53^. Finally, rTMS did not produce antinociception^42^ or affect the appetitive drive^56^.

### Risk of bias

**Fig. 5:**
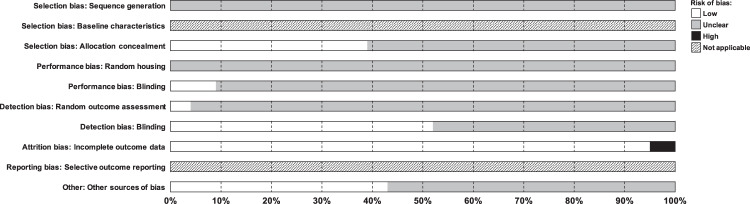
Risk of bias assessment, score (%) per risk of bias item. The RoB tool for animal studies contains 10 entries related to selection bias, performance bias, detection bias, attrition bias, reporting bias and other biases. For each entry, signaling questions were formulated to facilitate judgment^34^: “yes” indicates low risk of bias, “no” indicates high risk of bias, and “unclear” indicates an unclear risk of bias. If one of the relevant signaling questions is answered with “no,” this indicates high risk of bias for that specific entry. Assessing reporting bias was judge as “not applicable” for 2 items. In this respect it should be noted that the “Reporting bias” item was prospectively included in the SYRCLE’s tool (in agreement with the Cochrane’s tool) although at present difficult to assess, as protocols for animal studies are not yet mandatorily registered in central, publicly accessible databases^34,99^.

The risk of bias assessment of all included studies is shown in Fig. 5. Reporting of experimental details in animal studies is often poor (e.g.,^61^) and, consequently, studies had an overall unclear risk of bias based on SYRCLE’s RoB tool (55.3%). For 2 instances (20%), assessing reporting bias was judge as “not applicable”^34^. When not unclear, the risk of bias was generally low (24.2%), with the only exception of one study for the item “Attrition bias” (0.5%).

## Discussion

To our knowledge, this is the first systematic review and meta-analysis aiming to evaluate rTMS efficacy in preclinical models of depression. Overall, results show a largely beneficial effect of active rTMS compared to sham stimulation, as reflected in the statistically significant decrease in depressive-like symptoms. Most studies used stress-induced depression models (i.e., CUS, an antidepressant-sensitive depression model^22^). As for rTMS treatment characteristics, parameters varied considerably in terms of frequency, intensity and duration.

Notwithstanding the paucity and heterogeneity of studies, results are generally consistent. As expected, an overt depressive-like phenotype (i.e., CUS model) was more likely to be associated with a larger effect size. The positive direction of the effect was consistent across studies for both helplessness and anhedonia, though with different magnitude. Robust results were obtained both in models of depression and healthy animals. The effectiveness of rTMS in healthy models compares with human studies involving non-depressed patients and healthy subjects. For example, left human DLPFC stimulation has been demonstrated to reverse hedonic tone dysfunction in addicted subjects^62^, as well as to induce a more pronounced sensitivity to rewarding stimuli in healthy subjects^63,64^. Also, rTMS appeared to selectively reverse depressive-like symptoms while effects on other domains (e.g., anxiety) were rather mixed. This differential effect suggests that a “pure” depressive phenotype may be a specific and meaningful clinical target of rTMS interventions. Present data confirm the relevance of rTMS use in MDD and give an indication as to which patient subtype may benefit the most (i.e., “pure” depression as opposed to anxious and/or agitated forms). Preliminary suggestions on possible synergistic (i.e., rTMS combined with quetiapine) and antagonizing (i.e., rTMS combined with CB1 receptor antagonist) interactions between neuromodulation and pharmacotherapy also emerge. Translational findings from future preclinical studies should investigate other augmenting (e.g., lithium) effects of pharmacological agents and help guide integrated (rTMS plus pharmacotherapy) approaches^65^.

Clinical translation of results is also significant in terms of the reversal of depressive-like symptomatology regardless of certain neuromodulation parameters (i.e., frequency and duration). Indeed, reversal of the depressive phenotype was obtained independently of rTMS frequency. This result may be of translational relevance, given that current clinical applications of rTMS include use at both high and low frequencies, though targets have a different lateralization. Accruing evidence indicates an imbalance between the left and right DLPFC in MDD, supporting the need for differentiated stimulation/inhibition lateralized protocols to counterbalance such asymmetry. Left-right DLPFC imbalance is associated with neuropsychological (i.e., negative emotional judgment^66^) and metabolic alterations (i.e., left/dominant glutamate/GABA-related motor cortex hypoexcitability^67^), both involved in MDD pathophysiology. The fact that up to now lateralization has not been possible in animal models is a major limitation of preclinical studies that hampers accurate translation and substantiation of the right-left prefrontal imbalance pathophysiological hypothesis^66^. To overcome the lack of specificity of stimulation, coils optimized for precise targets—specifically designed for preclinical application—are needed^19^. Hopefully, technological advances and increased interest towards rTMS in animal models will allow the development of smaller sized coils for isolated stimulation of specific regions^38^. The availability of optimized coils will also allow to extend the investigation to smaller species (the presence of only 2 studies employing mice should be considered as a limitation).

When helplessness studies were grouped according to frequency, moderate to high heterogeneity persisted for high frequency studies, while for low frequency studies heterogeneity was remarkably lower. Similarly, after application of model-based subgroup analysis, the heterogeneity was considerably lower for studies performed in healthy subjects, while there was still substantial evidence of high heterogeneity particularly between studies employing “other” models. This is likely due to the diversity of approaches used to induce depression (e.g., genetic^68,69^, lesion^59^, selective breeding^70,71^). Lack of studies assessing rTMS efficacy in the same type of model (the “other” subgroup comprises 6 studies in 6 different models) precluded the possibility to refine subgroup analysis. As for anhedonia, even though all studies employed the CUS model, there was still substantial evidence of high heterogeneity, which may be partly attributed to individual differences in rats’ hedonic status, as well as to differences in sucrose concentration (1% vs. 2%). It is worth noting that the duration of stress application (3 vs. 8 weeks) and the stress regime itself may also explain some of the discrepancies among studies^22^. A subgroup analysis could not be performed due to the paucity of studies.

Though rTMS appears to significantly reverse the depressive phenotype, results from the present meta-analysis do not allow us to draw conclusions on its relative efficacy on specific depression models due to the low representation of the different existing models (e.g., early life stress, social stress, genetically engineered rodents^20,21^). To date, only the CUS model is well-represented (8 out of the 11 studies included, the remaining employed 3 different and incomparable models). Models addressing the interaction between environmental and predisposing genetic factors in the induction of depressive-like phenotypes are still rarely employed but highly relevant as they resemble real-life clinical situations (e.g., paradigms superimposing poor maternal care or mild early life stress on 5-HTT knockout rodents^72,73^ or investigating the interaction between reduced 5-HT and increased glucocorticoids during early postnatal life^74,75^). Extending rTMS studies to other models of depression that involve different pathogenic mechanisms is therefore crucial to test its effectiveness and to inform on the biological basis of treatment response to rTMS.

Stress models of depression, while well-validated in preclinical research, encompass a broad array of symptoms thus possibly diluting specific target treatment effects and introducing bias when examining biological changes associated with rTMS. Narrower phenotypes may capture the biological effects of rTMS and improve prediction of treatment response^63^. The DSM-5 definition of MDD likely represents an aggregate of different and probably highly diverse disease subtypes, each of which should be studied independently and might require specific therapeutic strategies^76^. It is very unlikely that DSM-5 MDD symptoms cluster as a consequence of a single pathophysiological process, especially in light of accruing evidence indicating distinct endophenotypes (e.g., resting state neural networks, genetic profiles) for patients with different symptom profiles diagnosed as having MDD^77^. In this context, translating preclinical results into clinical practice requires the use of other validated models of depression (corresponding to distinct endophenotypes) and, whenever possible, of additional and/or more sophisticated tests to evaluate depressive-like symptomatology^78^. This will help identify diagnostic biomarkers that predict response to rTMS and develop more tailored interventions^79^. To date, only the FST is well-represented (for further details on how the readouts used to assess the effectiveness of rTMS were controlled for potential changes in locomotor activity see Supplementary item 5), a limitation that should be considered when interpreting the findings of this work^21^. In spite of the numerous controversies existing around its ability to reproduce behavioral despair/helplessness (e.g.,^80,81^), the FST remains one of the most widely used tests to screen antidepressant effects^82,83^.

Current animal models of depression, including CUS, respond to conventional antidepressants. Therefore, they can inform about the antidepressant-like efficacy of a novel intervention but have limited utility in predicting whether such intervention will also be effective in patients suffering from TRD. To overcome this issue, future studies should involve treatment-resistant animal models of depression^7^ (at present, there are no studies on the effects of rTMS in these models). CUS-exposed rodents that fail to respond to conventional antidepressants are considered non-responders^84,85^. Also, Wistar-Kyoto rats subjected to CUS have been recently validated as a model of TRD^86,87^. Notably, these models showed a good response to deep brain stimulation^84–87^; however, the effects of rTMS in these treatment-resistant animals have not yet been studied.

Sex differences in depressive-like symptomatology have been evidenced in several animal models (e.g., CUS, FSL) and tests (e.g., FST) and there are marked sex differences in the prevalence of MDD^88,89^. The presence of only 2 studies including female subjects should therefore be considered as a limitation. Preclinical experiments are often conducted only in males (and, when they do include both sexes, subgroup analyses are often not reported), while clinical trials include both men and women^90,91^. This issue may partly explain why clinical trials repeatedly fail to confirm the expected benefits of new treatment approaches that have shown favorable profiles in preclinical studies^92^. To maximize translation of research findings to the clinical practice, sex should be considered as an important biological variable from basic and preclinical research^90,91^. To overcome the issue of overlooked and underreported sex and gender in research across disciplines, the SAGER guidelines (Sex And Gender Equity in Research) were published in 2016^93^. Accordingly, numerous scientific journals are revising their editorial policies requiring clear reporting of the sex/gender of research subjects and to analyze data by sex^94^. Future studies should also test whether rTMS effects persist longitudinally and, if so, to what extent, as currently follow-up evaluations are rarely performed.

Studies had an overall unclear risk of bias based on SYRCLE’s RoB tool. Unfortunately, this confirms that reporting of methodological details in animal studies remains poor (e.g.,^61,94^), even after publication in 2010 of the ARRIVE guidelines (Animal Research: Reporting of In Vivo Experiments^95^), which were developed to improve the design, analysis and reporting of research using animals. Although ARRIVE guidelines are currently endorsed by numerous scientific journals and societies^96^, most animal research papers still fail to meet minimum reporting standards (e.g.,^97,98^). In particular, none of the 13 articles published after 2010 declares adherence to ARRIVE guidelines. However, in the absence of mandatory reporting standards for pre-clinical animal studies, we cannot assume that the authors conducted their experiment in an inappropriate way; therefore, studies were not excluded based on a poor-quality score. Nevertheless, there is an urgent need to overcome the issue of largely unclear risk of bias in animal studies by improving compliance with the ARRIVE guidelines as (i) poor reporting of animal research hinders the quality of research and its potential to translate into the clinic; (ii) the quality of a systematic review/meta-analysis is dependent on the quality of the included studies, and not knowing the actual risk of bias hampers our ability to draw reliable conclusions. An approach that may likely mitigate the reporting bias in preclinical studies is constituted by the possibility to prioritize manuscripts that were preceded by a pre-registration procedure analogous to systematic reviews^99^.

Notwithstanding its limitations, this meta-analysis supports the efficacy of rTMS interventions in ameliorating phenotypic alterations isomorphic to human MDD symptoms in laboratory rodents. Predictions of the effects of rTMS, such as changes on the molecular and cellular levels up to modulations of brain networks, need further investigation in order to reconcile the varying observations that have been made so far in the rTMS field^16^. Large scale, prospective, and well-designed animal studies are necessary to clarify which stimulation protocols (i.e., number of pulses, stimulation frequency and intersession pauses) maximize clinical effects and to develop cost-effective protocols, with the potential of yielding faster clinical responses (i.e., accelerated rTMS^100–102^). Further exploration of rTMS use in rodent models will promote data-driven identification of prognostically-informative depression endophenotypes to be used in real-world MDD treatment settings to predict which patients are more likely to respond to rTMS, thus working toward a patient-tailored intervention for MDD and TRD. This will have a major impact in terms of clinical burden reduction and decreased exposure to non-specific and ineffective treatments.

## Supplementary information

Supplementary captions

Supplementary item 1

Supplementary item 2

Supplementary item 3

Supplementary item 4

Supplementary item 5
