# Supplementary material for: Recovering from depression with repetitive transcranial magnetic stimulation (rTMS): a systematic review and meta-analysis of preclinical studies
Source: Transl Psychiatry. 2020 Nov 10;10:393. doi: 10.1038/s41398-020-01055-2 (PMC7655822; doi:10.1038/s41398-020-01055-2)
Supplement: Supplementary file 1 — Supplementary captions [file 41398_2020_1055_MOESM1_ESM.doc]

**Supplementary material captions**

**Supplementary item 1.** Registered protocol of the systematic review and meta-analysis based on SYRCLE’s format for animal intervention studies.

**Supplementary item 2.** Search strategies used in each database.

**Supplementary item 3.** Forest plot (effect size and 95% CI) of individual comparisons of animals receiving active (n=322 animals) vs. sham (n=232 animals) rTMS intervention on the helplessness profile (overall effect).

*Notes.* Horizontal lines represent 95% CIs. The area of each square is proportional to the study weight in the analysis. The diamond represents pooled estimates from random-effects meta-analyses. Red line represents the overall effect. Studies with multiple experimental groups (i.e., exposed to rTMS intervention with a different number of pulses per intervention or a different intensity) are split in multiple lines (indicated by a, b, c, etc.); these were considered as independent comparisons in the meta-analysis after correcting the total number of control animals by dividing the number of animals in the control group by the number of intervention groups served. *Abbreviations.* A: active rTMS intervention; S: sham rTMS intervention; SDM: standardized mean difference, SE: standard error, CI: confidence interval.

**Supplementary item 4.** Funnel plots overseeing publication bias of studies included in the meta-analyses. As precision of the estimated intervention effect increases as the size of the study increases, effect estimates from small studies are expected to be scatter more widely at the bottom of the graph, with the spread narrowing among larger studies. In the absence of publication bias the plot should approximately resemble a symmetrical (inverted) funnel. **a)** Funnel plot of standardized mean diﬀerences (SDMs) from 20 studies/29 independent comparisons (ﬁlled circles) on the effects of rTMS on the helplessness profile. **b)** Funnel plot of standardized mean diﬀerences (SDMs) from 7 studies/8 independent comparisons (ﬁlled circles) on the effects of rTMS on the anhedonic profile. The presence of publication bias reflects the fact that studies with small sample sizes are more likely to be published if they have larger than average effects, which makes them more likely to meet the criterion for statistical significance.

**Supplementary item 5.** Table illustrating the effects of rTMS intervention on the behavioral profile related to other domains (i.e., general activity, weight measurement, social interaction, etc.) on all available parameters within each test and at all timepoints (on-going, shorth-term, long-term).

*****Reduction in passive behavior (i.e. immobility/floating) is interpreted as an antidepressant-like effect of the manipulation, provided that it does not increase general activity, which may produce a false positive result in the FST. However, as a significant decrease in locomotor activity (indicative of psychomotor retardation) is induced by the CUS procedure, an increase in distance traveled in the OFT in the CUS model can be interpreted as a recovery of the depressive-like phenotype. To exclude potential confounding effects in the interpretation of the FST, comparisons with additional control groups are usually performed (i.e. non-depressed vs depressed subjects both exposed to the active rTMS intervention; e.g. ^57^).

*Notes.* Timing is referred to the rTMS intervention; recovery: recovery of the phenotype in animal models of disease. *Abbreviations.* OFT: open-field test; BW: bodyweight; SIT: social interaction test; NSFT: novelty-suppressed feeding test; ↓ ↑: statistically significant change; ns: not significant.
