# Supplementary material for: Recovering from depression with repetitive transcranial magnetic stimulation (rTMS): a systematic review and meta-analysis of preclinical studies
Source: Transl Psychiatry. 2020 Nov 10;10:393. doi: 10.1038/s41398-020-01055-2 (PMC7655822; doi:10.1038/s41398-020-01055-2)
Supplement: Supplementary file 2 — Supplementary item 1 [file 41398_2020_1055_MOESM2_ESM.pdf]

SYSTEMATIC REVIEW PROTOCOL FOR ANIMAL INTERVENTION STUDIES

FORMAT BY SYRCLE (WWW.SYRCLE.NL)
VERSION 2.0 (DECEMBER 2014)

Item	Section/Subsection/Item	Description
	A. General	
1.	Title of the review	Recovering from depression with repetitive transcranial magnetic stimulation (rTMS): a systematic review of preclinical studies
2.	Authors (names, affiliations, contributions)	Luisa De Risio[1]: conceived and designed the study, writing the first draft of the manuscript<br>Marta Borgi[2]: data analysis, writing the first draft of the manuscript<br>Mauro Pettorruso[3]: conceived and designed the study, writing the first draft of the manuscript<br>Andrea Miuli[3]: data extraction and analysis<br>Angela Maria Ottomana[2]: data extraction and analysis<br>Antonella Sociali[3]: data extraction and analysis<br>Giovanni Martinotti[3]: revising subsequent drafts, consolidating the manuscript and contributing to its final version<br>Giuseppe Nicolò[1]: revising subsequent drafts, consolidating the manuscript and contributing to its final version<br>Simone Macrì[2]: revising subsequent drafts, consolidating the manuscript and contributing to its final version<br>Massimo di Giannantonio[3]: revising subsequent drafts, consolidating the manuscript and contributing to its final version<br>Francesca Zoratto[2]: conceived and designed the study, data extraction and analysis, writing the first draft of the manuscript<br><br>Affiliations:<br>[1] Department of Psychiatry, ASL Roma 5, Colleferro (Rome), Italy<br>[2] Center for Behavioral Sciences and Mental Health, Istituto Superiore di Sanità, Rome Italy<br>[3] Department of Neuroscience, Imaging and Clinical Sciences, "G. d'Annunzio" University, Chieti, Italy
3.	Other contributors (names, affiliations, contributions)	None
4.	Contact person + e-mail address	Francesca Zoratto: francesca.zoratto@iss.it
5.	Funding sources/sponsors	This work was partly supported by the "Departments of Excellence 2018-2022" initiative of the Italian Ministry of Education, University and Research for the Department of Neuroscience, Imaging and Clinical Sciences (DNISC) of the University of Chieti-Pescara
6.	Conflicts of interest	None
7.	Date and location of protocol registration	The protocol was submitted to the PROSPERO registry on November 6th, 2019
8.	Registration number (if applicable)	Registration number: CRD42019157549 (November 29th, 2019)
9.	Stage of review at time of registration	Preliminary searches: Started<br>Piloting of the study selection process: Started<br>Formal screening of search results against eligibility criteria: Not yet started<br>Data extraction: Not yet started<br>Risk of bias (quality) assessment: Not yet started

		Data analysis: Not yet started
	**B. Objectives**	
	Background	
10.	What is already known about this disease/model/intervention? Why is it important to do this review?	Transcranial magnetic stimulation (rTMS) is recognized as a feasible and effective treatment intervention for major depression in human subjects. Recently, a translational application of rTMS in preclinical models has been developed. Translational studies have the potential to further neurobiological correlates of its application, as well as to set optimal protocols to improve the clinical application of neuromodulation in mood disorders. The present review aims to explore the efficacy of rTMS interventions in animal models of depression, in order to identify strength and limits of the translational application of neuromodulation.
	Research question	
11.	Specify the disease/health problem of interest	Major depression
12.	Specify the population/species studied	Rats and mice
13.	Specify the intervention/exposure	Active rTMS intervention
14.	Specify the control population	Sham rTMS intervention
15.	Specify the outcome measures	Variation of the depressive-like phenotype in subjects exposed to active rTMS compared with sham intervention
16.	State your research question (based on items 11-15)	Based on preclinical evidence, is rTMS intervention effective in reversing the depressive-like phenotype in animal models?
	**C. Methods**	
	Search and study identification	
17.	Identify literature databases to search (*e.g.* Pubmed, Embase, Web of Science)	☑MEDLINE via PubMed ☑Web of Science<br>☑SCOPUS ☐EMBASE<br>☐Other, namely:<br>☐Specific journal(s), namely:
18.	Define electronic search strategies (*e.g.* use the step by step search guide and animal search filters)	See the supplementary file containing the search strategy: "Search strategy.pdf"
19.	Identify other sources for study identification	☐Reference lists of included studies ☐Books<br>☐Reference lists of relevant reviews<br>☐Conference proceedings, namely:<br>☐Contacting authors/organizations, namely:<br>☐Other, namely:
20.	Define search strategy for these other sources	n/a
	Study selection	
21.	Define screening phases (*e.g.* pre-screening based on title/abstract, full text screening, both)	First phase: screening based on title and abstract; second phase: full-text screening of the eligible articles
22.	Specify (a) the number of reviewers per screening phase and (b) how discrepancies will be resolved	(a) Within each selection phase, two independent reviewers per article (AO and FZ); differences will be solved through discussion or by consulting additional investigators (MP and MB)
	Define all inclusion and exclusion criteria based on:	
23.	Type of study (design)	Inclusion criteria: No restrictions on the types of study design

		eligible for inclusion will be applied<br>Exclusion criteria: None
24.	Type of animals/population (*e.g.* age, gender, disease model)	Inclusion criteria: Rodent species (mice and rats), both sexes, all ages<br>Exclusion criteria: Studies in vitro, studies in humans, studies in non-human animals other than rodents
25.	Type of intervention (*e.g.* dosage, timing, frequency)	Inclusion criteria: Repetitive transcranial magnetic stimulation (rTMS) intervention<br>Exclusion criteria: Neuromodulation interventions other than rTMS (e.g. single pulse TMS; transcranial direct current stimulation, tDCS)
26.	Outcome measures	Inclusion criteria: Variation of the depressive-like phenotype reported<br>Exclusion criteria: Other outcome measures reported in the absence of an assessment of the depressive-like phenotype
27.	Language restrictions	Inclusion criteria: English language<br>Exclusion criteria: Language other than English
28.	Publication date restrictions	Inclusion criteria: All publication dates<br>Exclusion criteria: None
29.	Other	Type of control intervention:<br>Inclusion criteria: Sham-treated animals (i.e. animals exposed to the sham rTMS intervention)<br>Exclusion criteria: Non-treated animals (i.e. animals not exposed to the sham rTMS intervention)<br><br>Other:<br>Inclusion criteria: Articles presenting original researches, full-text articles<br>Exclusion criteria: Non-original researches (e.g. reviews, commentaries, editorials, book chapters), no full-text articles (e.g. meeting abstracts)
30.	Sort and prioritize your exclusion criteria per selection phase	1. Language other than English<br>2. Non-original researches (e.g. reviews, commentaries, editorials, book chapters)<br>3. No full-text articles (e.g. meeting abstracts)<br>4. Studies in vitro, studies in humans, studies in non-human animals other than rodents<br>5. Other outcome measures reported (e.g. anxiety, general activity, body weight) in the absence of an assessment of the depressive-like phenotype<br>6. Neuromodulation interventions other than rTMS (e.g. single pulse TMS; transcranial direct current stimulation, tDCS)<br>7. Animals not exposed to the sham rTMS intervention as comparator/control<br>The prioritization applies to both selection phases (i.e. screening based on title and abstract; full-text screening of the eligible articles)
	Study characteristics to be extracted	
31.	Study ID (*e.g.* authors, year)	Title, authors, publication year, journal
32.	Study design characteristics (*e.g.* experimental groups, number of animals)	Number of experimental groups, number of subjects per group, type of study design (i.e. within- vs between-subjects)

33.	Animal model characteristics (*e.g.* species, gender, disease induction)	Species, strain, sex, age and/or weight at the beginning of the study, type of model employed (e.g. animal models of depression, healthy animal models, models other than depression), type of tests used to evaluate the depressive-like phenotype and the other relevant behavioral phenotypes (including timing), biological correlates investigated (when applicable)
34.	Intervention characteristics (*e.g.* intervention, timing, duration)	Neurostimulation intervention: frequency (Hz), pulses per train, number of trains, pulses per session, number of sessions, total pulses, intensity (% RMT or Tesla), train duration (s), inter-train interval (s), session duration (min), inter-session interval (h), coil type, area stimulated, use of anesthesia<br>Pharmacological intervention (when applicable): substance, general properties, dosage, administration route, duration of treatment
35.	Outcome measures	Difference in the depressive-like phenotype (e.g. recovery vs deterioration) and in other behavioral phenotypes relevant to depression (i.e. anxiety, general activity, body weight) between active vs sham rTMS intervention
36.	Other (*e.g.* drop-outs)	Augmenting or antagonizing effects of the concomitant administration of rTMS and a pharmacological agent on the depressive-like phenotype
	Assessment risk of bias or study quality	
37.	Specify (a) the number of reviewers assessing the risk of bias/study quality in each study and (b) how discrepancies will be resolved	(a) The criteria will be independently assessed by two reviewers (AO and AM); (b) differences of opinion that cannot be resolved by discussion will be solved by consulting additional investigators (FZ and MP)
38.	Define criteria to assess (a) the internal validity of included studies (*e.g.* selection, performance, detection and attrition bias) and/or (b) other study quality measures (*e.g.* reporting quality, power)	☑By use of SYRCLE's Risk of Bias tool<br>☐By use of SYRCLE's Risk of Bias tool, adapted as follows:<br>☐By use of CAMARADES' study quality checklist<br>☐By use of CAMARADES' study quality checklist, adapted as follows:<br>☐Other criteria, namely:
	Collection of outcome data	
39.	For each outcome measure, define the type of data to be extracted (*e.g.* continuous/dichotomous, unit of measurement)	The direction of the variation (i) of the depressive-like phenotype (e.g. recovery vs deterioration, including the augmenting or antagonizing effects of concomitant pharmacological interventions), and (ii) of other behavioral phenotypes relevant to depression (i.e. anxiety, general activity, body weight) will be retrieved<br>Depression/anhedonia:<br>- sucrose preference ratio in the sucrose preference test (SPT), continuous data, unit of measurement: n/a (dimensionless number)<br>- sucrose intake in the sucrose consumption test (SCT), continuous data, unit of measurement: milliliter (or other unit of volume)<br>Depression/helplessness:<br>- immobility duration in the forced swim test (FST), continuous data, unit of measurement: seconds<br>Anxiety:<br>- time in the center of the arena in the open-field test (OFT), continuous data, unit of measurement: seconds<br>- latency to feed in the novelty-suppressed feeding test (NSFT),

		continuous data, unit of measurement: seconds<br>General activity:<br>- distance traveled in the open-field test (OFT), continuous data, unit of measurement: cm (or other unit of distance)<br>Weight measurement:<br>- body weight, continuous data, unit of measurement: grams
40.	Methods for data extraction/retrieval (*e.g.* first extraction from graphs using a digital screen ruler, then contacting authors)	Data will be extracted from graphs using a digital screen ruler
41.	Specify (a) the number of reviewers extracting data and (b) how discrepancies will be resolved	(a) Data regarding the animal model will be independently extracted by AO and FZ, data regarding the stimulation parameters will be independently extracted by AM and AS; (b) discrepancies that cannot be resolved by discussion will be solved by consulting additional investigators (MP and MB)
	Data analysis/synthesis	
42.	Specify (per outcome measure) how you are planning to combine/compare the data (*e.g.* descriptive summary, meta-analysis)	A formal narrative (descriptive) synthesis is planned; a quantitative synthesis (meta-analysis) will be considered if a group of studies will be sufficiently homogeneous (same type of intervention and comparator, with the same outcome measure) to provide a meaningful summary
43.	Specify (per outcome measure) how it will be decided whether a meta-analysis will be performed	Meta-analyses and subgroup analyses will be performed for each depressive-like outcome measure (i.e. anhedonia and helplessness) reported in 8 or more independent comparisons
	If a meta-analysis seems feasible/sensible, specify (for each outcome measure):	
44.	The effect measure to be used (*e.g.* mean difference, standardized mean difference, risk ratio, odds ratio)	Effect size calculations will be based on a comparison between the treated group (receiving the active rTMS intervention) and the control group (receiving the sham intervention); we will provide summaries of intervention effects for each individual treated-control comparison by calculating the Standardized Mean Difference (SMD); the individual SMDs will be pooled to obtain an overall SMD and 95% confidence interval
45.	The statistical model of analysis (*e.g.* random or fixed effects model)	In order to take into account the anticipated heterogeneity, a random-effect model will be used to compute both the overall effect size and the separate effect sizes for the different subgroups
46.	The statistical methods to assess heterogeneity (*e.g.* I^2, Q)	The I^2 statistic will be used as a measure of heterogeneity between studies
47.	Which study characteristics will be examined as potential source of heterogeneity (subgroup analysis)	Should the necessary data be available, we plan to perform subgroup analyses by rTMS intervention's frequency (e.g. high: >5Hz, low: ≤5Hz) and by type of animal model (e.g. model of disease, healthy model)
48.	Any sensitivity analyses you propose to perform	Potential sources of methodological diversity will be taken into account; specifically, in the presence of one or two studies presenting characteristics that render them different from the others, a sensitivity analysis will be performed excluding those studies from the meta-analysis<br>Sensitivity analyses will be also performed by repeating the primary meta-analysis, substituting alternative decisions or ranges of values for decisions that may be considered arbitrary (e.g. high frequency: ≥5Hz, low frequency: <5Hz)
49.	Other details meta-analysis (*e.g.*	Whenever a control group serves more than one experimental

	correction for multiple testing, correction for multiple use of control group)	group, we will correct the total number of control animals in the meta-analysis by dividing the number of animals in the control group by the number of intervention groups served
50.	The method for assessment of publication bias	A funnel plot of study effect sizes against standard errors will be visually inspected for asymmetry; asymmetry will be also tested statistically with Egger’s bias test with $p<0.05$ indicating asymmetry

Final approval by (names, affiliations):
Luisa De Risio[1], Marta Borgi[2], Mauro Pettorruso[3], Andrea Miuli[3], Angela Maria Ottomana[2], Antonella Sociali[3], Giovanni Martinotti[3], Giuseppe Nicolò[1], Simone Macrì[2], Massimo di Giannantonio[3], Francesca Zoratto[2]

Affiliations:
[1] Department of Psychiatry, ASL Roma 5, Colleferro (Rome), Italy
[2] Center for Behavioral Sciences and Mental Health, Istituto Superiore di Sanità, Rome Italy
[3] Department of Neuroscience, Imaging and Clinical Sciences, "G. d'Annunzio" University, Chieti, Italy

Date: 02-12-2019
