# Supplementary material for: Recovering from depression with repetitive transcranial magnetic stimulation (rTMS): a systematic review and meta-analysis of preclinical studies
Source: Transl Psychiatry. 2020 Nov 10;10:393. doi: 10.1038/s41398-020-01055-2 (PMC7655822; doi:10.1038/s41398-020-01055-2)
Supplement: Supplementary file 3 — Supplementary item 2 [file 41398_2020_1055_MOESM3_ESM.pdf]

Search strategy

SCOPUS

By using the field “TITLE-ABS-KEY” we will search in title, abstract and keywords (both author and indexed keywords).

Search string: (TITLE-ABS-KEY ("depression") AND TITLE-ABS-KEY ("repetitive transcranial magnetic stimulation" OR "rTMS") AND TITLE-ABS-KEY ("rat" OR "rats" OR "mouse" OR "mice"))

PUBMED

By using the field “Title/Abstract” we will search in title, abstract and keywords (author keywords).

Search string: (("depression"[Title/Abstract]) AND ("repetitive transcranial magnetic stimulation"[Title/Abstract] OR "rTMS"[Title/Abstract])) AND ("rat"[Title/Abstract] OR "rats"[Title/Abstract] OR "mouse"[Title/Abstract] OR "mice"[Title/Abstract])

WEB OF SCIENCE

By using the field “TS” we will search in title, abstract and keywords (both author keywords and keywords plus).

Search string: ((depression) AND ((repetitive transcranial magnetic stimulation) OR (rTMS)) AND (rat OR rats OR mouse OR mice))
