# Supplementary figures and images for: Recovering from depression with repetitive transcranial magnetic stimulation (rTMS): a systematic review and meta-analysis of preclinical studies
Source: Transl Psychiatry. 2020 Nov 10;10:393. doi: 10.1038/s41398-020-01055-2 (PMC7655822; doi:10.1038/s41398-020-01055-2)

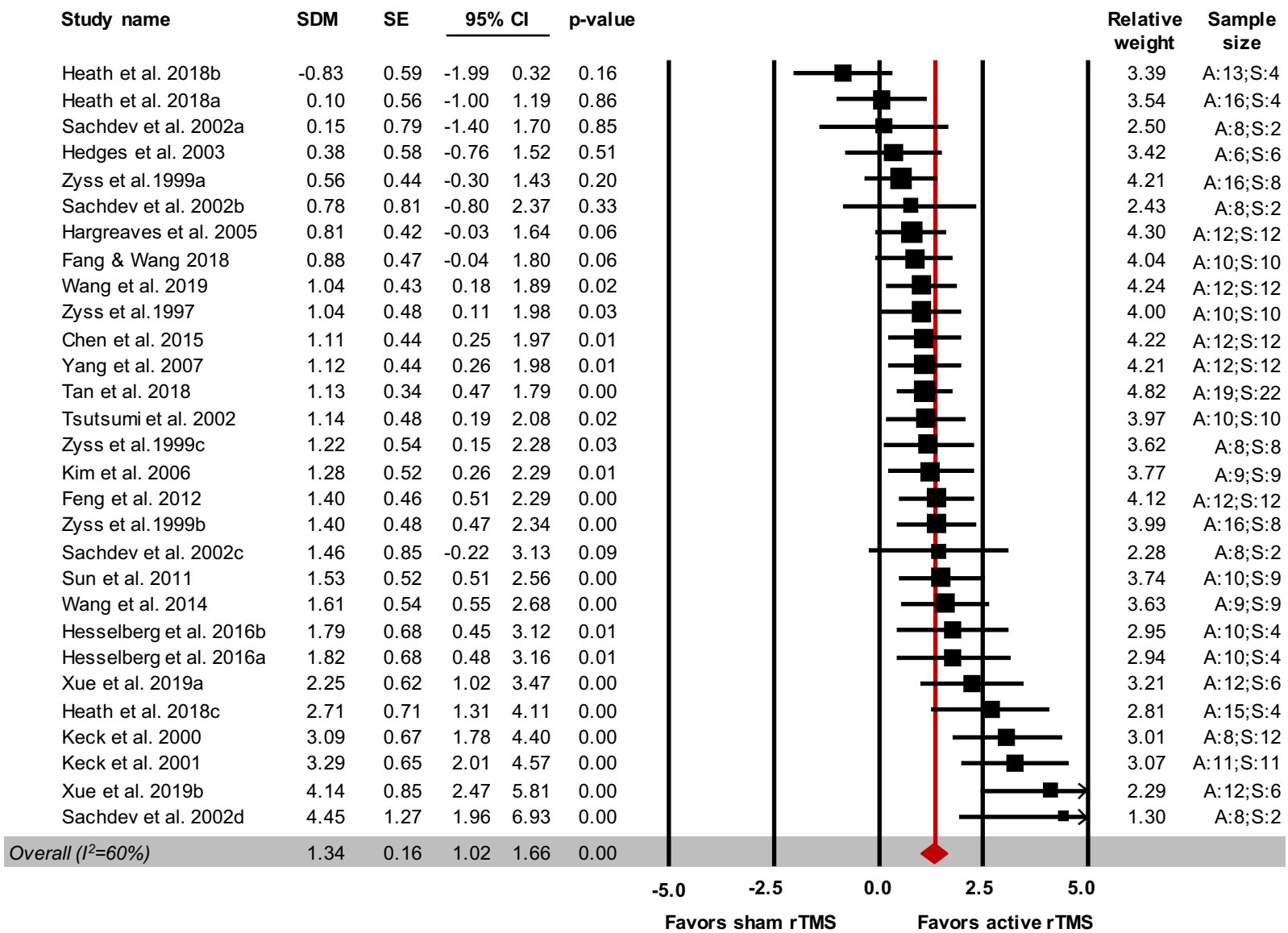

Supplement: Supplementary file 4 — Supplementary item 3 [file 41398_2020_1055_MOESM4_ESM.pdf]

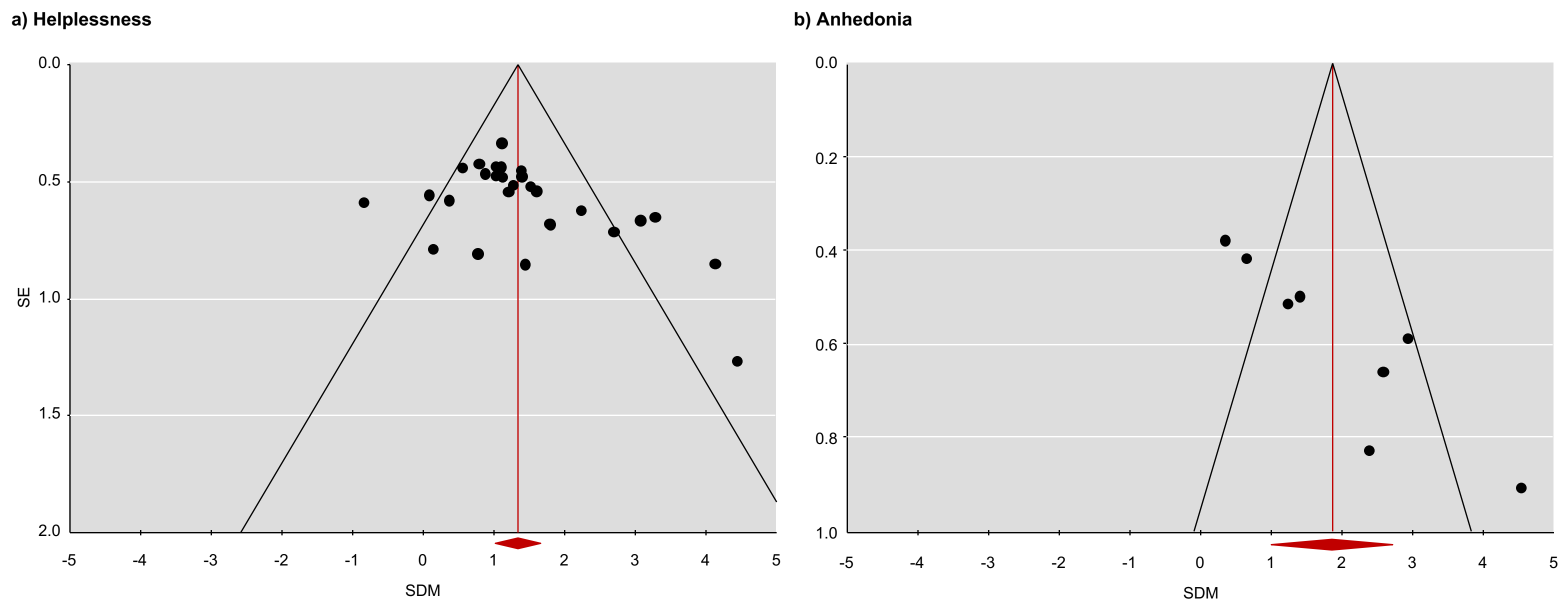

Supplement: Supplementary file 5 — Supplementary item 4 [file 41398_2020_1055_MOESM5_ESM.pdf]
