# Supplementary material for: Recovering from depression with repetitive transcranial magnetic stimulation (rTMS): a systematic review and meta-analysis of preclinical studies
Source: Transl Psychiatry. 2020 Nov 10;10:393. doi: 10.1038/s41398-020-01055-2 (PMC7655822; doi:10.1038/s41398-020-01055-2)
Supplement: Supplementary file 6 — Supplementary item 5 [file 41398_2020_1055_MOESM6_ESM.pdf]

Supplementary item 5. rTMS effects on the behavioral profile related to other domains. Study-wise list of dependent variables and results.

Study	Intervention duration	Test: parameter(s) used	1st assessment		2nd assessment	
			Timing	Results	Timing	Results
**General activity**						
Feng et al. 2012 [56]	3 weeks	OFT: distance traveled (mm)	During the 1st week after the intervention	**15Hz:** ns	During the 3rd week after the intervention	**15Hz:** ns
Hesselberg et al. 2016 [45]	10 days	OFT: distance traveled (cm)	24/48h after the last session	**1Hz:** ns; **20Hz:** ns		
Peng et al. 2018 [36]	7 days	OFT: distance traveled (cm)	24h after the last session	**1Hz (0.84 T):** ns; **1Hz (1.26 T):** ns; **5Hz (0.84 T):** ns; **5Hz (1.26 T):** ns; **10Hz (0.84 T):** ns; **10Hz (1.26 T):** ns		
Wang et al. 2014 [57]	7 days	OFT: distance traveled (mm)	24h after the last session	**15Hz:** ↑*		
Wang et al. 2019 [40]	2 weeks	OFT: distance traveled (cm)	8 days after the first session	**0.5Hz:** ns	24h after the last session	**0.5Hz:** ↑*
		OFT: frequency of rearing		**0.5Hz:** ns		**0.5Hz:** ↑*
		OFT: velocity (cm/s)		**0.5Hz:** ns		**0.5Hz:** ↑*
Xue et al. 2019 [58]	7 days	OFT: distance traveled (mm)	24/72h after the last session	**1Hz:** ns; **5Hz:** ns		
Zhao et al. 2018 [53]	3 weeks	OFT: distance traveled (mm)	Immediately after the last session	**10Hz:** ↑*		
		OFT: total vertical number		**10Hz:** ↑*		
Zyss et al. 1997 [42]	2 weeks	Locomotor activity: early phase exploratory activity (count)	2h after the last session	**50Hz:** ns		
		Locomotor activity: late phase basal activity (count)		**50Hz:** ↓		
**Weight measurement**						
Kim et al. 2014 [52]	2 weeks	BW: weight (g)	During the 1st week of intervention	**10Hz:** ↑ (=recovery)	During the 2nd week of intervention	**10Hz:** ↑ (=recovery)
Zhao et al. 2018 [53]	3 weeks	BW: weight reduction rate (%)	Immediately after the last session	**10Hz:** ↑ (=recovery)		
**Social interaction**						
Hargreaves et al. 2005 [37]	18 days	SIT: social interaction duration (s)	10/11 days after the first session	**20Hz:** ns		
		SIT: aggressive behavior duration (s)		**20Hz:** ns		
		SIT: escape activity duration (s)		**20Hz:** ns		
Keck et al. 2000 [46]	5 weeks	SIT: active interaction duration (s)	During the 2nd week after the intervention	**20Hz:** ns		

Tan et al. 2018 [51]	2 weeks	Three-chamber test: ratio of time spent in the rat/object compartment	24h after the last session	**1Hz:** ↑ (=recovery)		
**Appetitive drive**						
Feng et al. 2012 [56]	3 weeks	NSFT: amount of food consumed (g)	During the 1st week after the intervention	**15Hz:** ns	During the 3rd week after the intervention	**15Hz:** ns
**Stereotyped behaviors**						
Tan et al. 2018 [51]	2 weeks	Self-grooming in an open-field arena: duration of self-grooming (s)	48h after the last session	**1Hz:** ↓ (=recovery)		
**Analgesia**						
Zyss et al. 1997 [42]	2 weeks	Tail-flick test: latency of tail-flick response (s)	2h after the last session	**50Hz:** ns		

Table illustrating the effects of rTMS intervention on the behavioral profile related to other domains (i.e., general activity, weight measurement, social interaction, etc.) on all available parameters within each test and at all timepoints (on-going, shorth-term, long-term).
*****Reduction in passive behavior (i.e. immobility/floating) is interpreted as an antidepressant-like effect of the manipulation, provided that it does not increase general activity, which may produce a false positive result in the FST. However, as a significant decrease in locomotor activity (indicative of psychomotor retardation) is induced by the CUS procedure, an increase in distance traveled in the OFT in the CUS model can be interpreted as a recovery of the depressive-like phenotype. To exclude potential confounding effects in the interpretation of the FST, comparisons with additional control groups are usually performed (i.e. non-depressed vs depressed subjects both exposed to the active rTMS intervention; e.g. [57]).
Notes. Timing is referred to the rTMS intervention; recovery: recovery of the phenotype in animal models of disease. *Abbreviations.* OFT: open-field test; BW: bodyweight; SIT: social interaction test; NSFT: novelty-suppressed feeding test; ↓ ↑: statistically significant change; ns: not significant.
